# Correlations between omega-3 fatty acids and inflammatory/glial abnormalities: the involvement of the membrane and neurotransmitter dysfunction in schizophrenia

**DOI:** 10.3389/fncel.2023.1163764

**Published:** 2023-10-23

**Authors:** Yongping Zhang, Jingwen Yin, Haifeng Yan, Ling Yan, Yuyu Li, Cai Zhang, Yajuan Li, Baiping Liu, Juda Lin, Liqiang Zhang, Xueqiong Hu, Cai Song

**Affiliations:** ^1^Research Institute for Marine Drugs and Nutrition, College of Food Science and Technology, Guangdong Ocean University, Zhanjiang, China; ^2^Marine Medicine Research and Development Center of Shenzhen Institutes of Guangdong Ocean University, Shenzhen, China; ^3^Guangdong Provincial Key Laboratory of Aquatic Product Processing and Safety, College of Food Science and Technology, Guangdong Ocean University, Zhanjiang, China; ^4^Affiliated Hospital of Guangdong Medical University, Zhanjiang, China; ^5^Zhanjiang Third People's Hospital, Zhanjiang, China

**Keywords:** schizophrenia (SZ), inflammation, cytokines, polyunsaturated fatty acids (PUFAs), neurotransmitters

## Abstract

**Introduction:**

Macrophages or T-lymphocytes triggered inflammation and, consequently, activated glial cells may contribute to neuroinflammation and neurotransmitter dysfunction in schizophrenia (SZ), while omega(n)-3 polyunsaturated fatty acids (PUFAs) can attenuate some SZ symptoms through anti-inflammatory effects. However, the correlations between macrophage/T-lymphocyte-produced cytokines and glia phenotypes, between inflammatory status and PUFAs composition, between cytokines and neurotransmitter function, and between n-3 PUFAs and neurotransmitter abnormality in SZ are unclear.

**Methods:**

Changes in T-helper (h) patterns, peripheral macrophage/glial markers, PUFAs profile, membrane fluidity, and neurotransmitter functions were evaluated in SZ patients (*n* = 50) and healthy controls (*n* = 30) using ELISA, gas chromatography, fluorescence anisotropy techniques, and HPLC, respectively.

**Results:**

Compared to the control, blood lymphocyte proliferation, the concentration of macrophage/microglia phenotype M1 markers, including cytokines IL-1β, TNF-α (Th1) and IL-6 (Th2), and astrocyte phenotype A1 marker S100β was significantly increased, while IL-17 and n-3 PUFAs contents, n-3/n-6 ratio, and membrane fluidity (FLU) were significantly decreased in SZ. Moreover, increased DA and HVA, decreased 5-HT and NE, and their metabolites appeared in SZ. Moreover, negative correlations between IL-6 and A2 marker Brain-Derived Neurotrophic Factor (BDNF) or n-3 PUFAs EPA and between IL-1β and FLU or 5HIAA, while positive correlations between EPA and 5-HIAA and between FLU and DHA were found in SZ.

**Discussion:**

These findings showed (1) no clear Th pattern, but pro-inflammatory-dominant immunity occurred; (2) the pro-inflammatory pattern may result in the activated microglia M1 and astrocyte A1 phenotype; and (3) increased pro-inflammatory cytokines were related to decreased n-3 PUFA and decreased membrane fluidity and dysfunctional neurotransmitter systems in SZ.

## 1. Introduction

Dysfunction of monoamine neurotransmission, such as the dopamine system, was believed to contribute to schizophrenia (SZ) (Howes and Kapur, 2009; Purves-Tyson et al., 2017). However, increasing evidence suggests that inflammatory and autoimmune disorders play an important role in the etiology of SZ (Ganguli et al., 1994; Boerrigter et al., 2017; Purves-Tyson et al., 2020). Among many immune changes in SZ, the change in T-helper (h) cells and their released cytokines attracted great attention. As early as 2001, Schwarz et al. reported a shift from Th-1 response toward Th-2 response in SZ (Schwarz et al., 2001). However, changes in the Th1 cytokine pattern, such as interleukin (IL)-2 and interferon (IFN)-γ, in SZ were controversially reported in different studies (Cazzullo et al., 2001; Kim et al., 2009). Recently, IL-17 produced by Th17 was found to promote the Th-1 cell response by modulating dendritic cell function (Gottenberg and Chiocchia, 2007). Thus, changes in cytokine patterns of Th1, Th2, and Th17 in SZ should be further determined.

More interesting, Louveau et al. have recently reported the existence of lymphatics and lymphocytes in the brain (Tavares and Louveau, 2021), which raises a new research direction of what the role of lymphocyte subtypes and their released cytokines is in SZ. Our recent study has reported that imbalances between Th1/Th2 and Th17/Treg activities and their produced cytokines significantly contribute to glial dysfunction in a rodent model of depression (Huang et al., 2022). As the main innate immune cells in the brain, both microglia and astrocytes affect neuronal survival environment by secreting cytokines and neurotrophic factors. A plethora of studies have shown structural and functional abnormalities of both glia in SZ. On the one hand, increased densities of microglial cells and aberrant expression of microglia-related markers were found in schizophrenia (Bernstein et al., 2015). On the other hand, astrocyte activation is associated with immune changes in schizophrenia (Kolomeets and Uranova, 2014). It has been found that an increased astrocyte phenotype A1 marker S100β occurs in the plasma of SZ patients (Rothermundt et al., 2009). With regard to the relationship between T lymphocytes and glial cells, Th-1 releasing cytokines, IFN-γ and IL-2, mainly mediate the activation of microglia phenotype M1, which releases proinflammatory cytokines and mediate astrocyte phenotype A1. A1 can trigger neuroinflammatory response and neurodegeneration. By contrast, Th-2 releasing cytokines (IL-4, IL-6, and IL-10) activate microglia M2 (IL-10) and astrocyte A2 phenotype (BDNF), which tend to promote anti-inflammatory response and neuroprotection (Ma et al., 2015). Indeed, increased M1-like inflammatory response and A1 marker expression, but decreased anti-inflammatory mediators and BDNF were found in SZ (Gama et al., 2007; Reus et al., 2015; Kozlowska et al., 2021). However, the relationships between pro- or anti-inflammatory cytokines and microglia or astrocyte phenotypes in SZ are still unknown. Excessive neuroinflammation occurs in psychiatric diseases, including SZ, and may result from peripheral inflammatory diseases, malnutrition, and /or environmental pollution. Many studies, including ours, have recently demonstrated that diet/dietary factors, especially lipids play an important role in inflammatory response and neuroinflammation. It was found that essential omega (n)-3 polyunsaturated fatty acids (PUFAs) can prevent or treat depression and Alzheimer's disease by regulating membrane function and inhibiting neuroinflammation (Giacobbe et al., 2020; Liu et al., 2021). By contrast, a diet short of n-3 PUFAs may contribute to excessive inflammatory response and mental diseases (Beydoun et al., 2015; Larrieu and Laye, 2018). In schizophrenia patients, including both those treated with antipsychotic medication and antipsychotic-naive patients, reduced levels of n-3 PUFAs in the plasma and erythrocytes were reported (Sumiyoshi et al., 2011; van der Kemp et al., 2012). However, the correlation between n-3 PUFAs composition and Th or macrophages-produced cytokines remains unclear.

As mentioned above, inflammation is one of the causes of neurotransmitter dysfunction in SZ. Excessive inflammatory responses were found in both the peripheral and central immune system, such as macrophage and microglia M1 activation, which may result in neuroinflammation and hyper-activity of dopamine and glutamate system in SZ patients (Meyer et al., 2008; Ramirez-Jirano et al., 2019). Moreover, inflammation was found to increase mesencephalic progenitor cells and transform them into dopaminergic neurons in rats (Potter et al., 1999), which leads to increased release of dopamine. In PUFAs, eicosapentaenoic acid (EPA) in the n-3 series is a natural anti-inflammatory fatty acid. Another n-3 PUFA docosahexaenoic acid (DHA) deficiency has been associated with neuronal membrane instability and abnormal transmission of serotonin, norepinephrine, and dopamine, as well as associated with mood and cognitive impairment (Su et al., 2003; Chalon, 2006). However, the relationship between cytokine patterns and neurotransmitter dysfunction or the correlation between membrane n-3 PUFA levels and neurotransmitter dysfunction was not fully understood in SZ.

Furthermore, due to the different positions of double bonds and configurations of PUFAs, changes in membrane PUFAs directly affect membrane fluidity and function, which are related to gene and protein interaction, ion channel function, and neurotransmitter release and receptor bindings (Joensuu et al., 2020; Diaz et al., 2023). Decreased n-3 PUFAs may inevitably affect membrane fluidity in SZ. Therefore, the relationships between membrane fluidity and PUFA composition, inflammation, or neurotransmitter function in SZ also need to be clarified.

**Figure 1:**
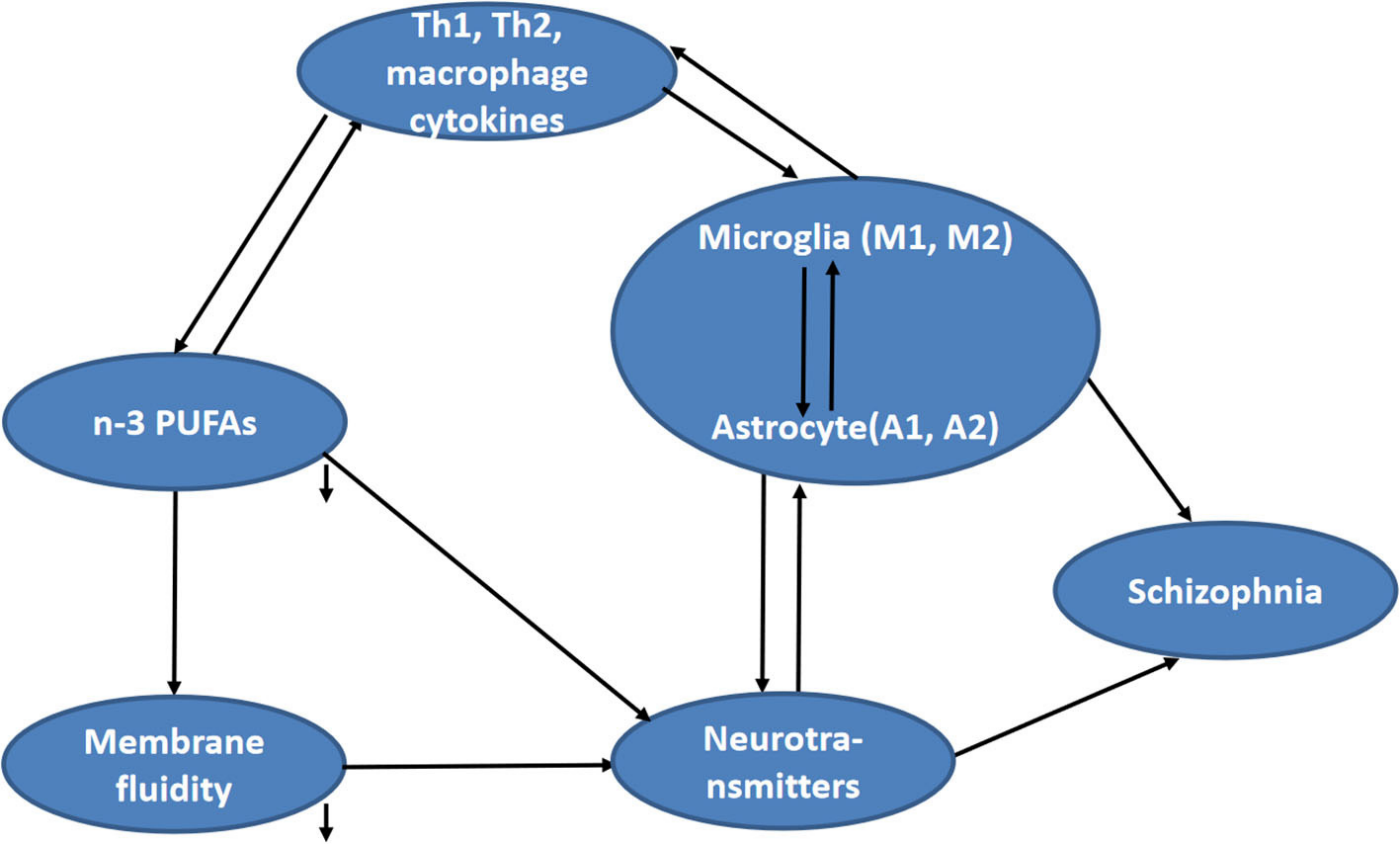
Hypothesized relationship between fatty acid composition and other concerned factors (including cytokines, neurotransmitter function, and membrane fluidity) in schizophrenia.

To explore the abovementioned five unknowns in schizophrenia, the present study measured lymphocyte activation, Th cell and glial phenotype markers, PUFA composition, membrane fluidity, and the concentration of monoamine neurotransmitters and metabolites in blood samples of schizophrenics and healthy controls. Then, the correlations between cytokines and glia phenotypes, between cytokines and PUFAs composition, between cytokines and neurotransmitter function, between n-3 PUFAs and neurotransmitter function, as well as between membrane fluidity and inflammation or neurotransmitters, were analyzed (Figure 1).

## 2. Methods

### 2.1. Subjects and study design

This is a case–control study with 50 SZ patients aged between 21 and 64 years from Zhanjiang Third People's Hospital in Zhanjiang, Guangdong, China, from March 2016 to March 2017, and 30 gender and age-matched healthy controls (recruited from near the hospital, mainly hospital staff and university students). Both subjects ethnically belonged to the Han nationality. All patients underwent illness assessment and blood collection within 2 weeks of admission after an acute attack. Patients were diagnosed as per the Chinese version of the International Classification of Diseases (ICD)-10/F20 criteria for schizophrenia by two psychiatrists. Data regarding tobacco, alcohol, and illicit drug use in the past 3 months were collected using a substance use questionnaire. Patients with major affective disorders, schizo-affective disorder, and narcotic drugs or alcohol abuse were excluded. Smoking is not allowed during hospitalization. Antipsychotics (type and dose) are shown in Table 1. To avoid medication-induced weight gain, hospital-controlled diets were provided and physical exercises were arranged. For some patients with significant weight gain after taking certain anti-psychotic medicines, the medication was changed. All the participants included consented to join the study. Healthy people without a family history of mental illness and a history of central nervous system diseases were selected as controls (Table 1). In the study, 5–10 ml of fasting peripheral blood was collected and used for cytokine, membrane lipid, and neurotransmitter analyses. The study was approved by the Research Ethics Committee for Biomedical Subjects according to Zhujiang Hospital of South Medical University guidelines [(2016) REC No. (026)].

**Table 1:** Demographic and clinical information of schizophrenic and healthy controls.

	***N* (schizophrenia/control)**	**Schizophrenia**	**Control**	***t* value/χ^2^**	***p*-value**
Age (years)	50/30	39.6 ± 11.7	40.2 ± 8.0	0.23	0.82
Gender (M/F)	50/30	25/25	15/15	0	1
Body mass index (BMI, kg/m^2^)	50/30	19.8 ± 2.5	19.9 ± 2.6	0.11	0.92
Education (years)	50/30	10.9 ± 1.6	12.0 ± 3.6	1.89	0.06
Smoking	48/28	12	2	3.85	0.05
Age of illness onset (years)	50/30	23.8 ± 8.7	-		-
Duration of illness (years)	50/30	11.7 ± 7.5	-		-
**BPRS score**					
Anxiety and depression	50/NA	8.5 ± 4.1			
Lack of vitality	50/NA	8.4 ± 3.2			
Thinking disorders	50/NA	10.8 ± 4.2			
Activation	50/NA	7.1 ± 2.5			
Hostile suspicion	50/NA	11.0 ± 3.9			
**Antipsychotic medications (mg/day)**					
Olanzapine (mg/day)	13/NA	24.2 ± 22.9			
Clozapine (mg/day)	13/NA	182.7 ± 75.9			
Quetiapine (mg/day)	20/NA	565.0 ± 220.7			
Risperidone (mg/day)	7/NA	3.4 ± 1.5			
Sulpiride (mg/day)	4/NA	400 ± 163.3			
**Other medications (mg/day)**					
Sodium valproate	4/NA	800 ± 326			
Sertraline	4/NA	112.5 ± 62.9			
Buspirone	1/NA	10			
Clomipramine hydrochloride	1/NA	150			
Diazepam	4/NA	6.25 ± 2.5			
Lorazepam	3/NA	1.33 ± 0.6			
Estazolam	2/NA	2 ± 0			

M, male; F, female; BPRS, Brief Psychiatric Rating Scale; N/A, not available. Means or numbers provided. Standard deviation in parentheses.

### 2.2. General comments regarding methods

It has been reported that peripheral monocyte count and cytokine levels can be considered as an indirect marker of microglia activity or immune abnormality in the brain of schizophrenia disorder (Bergink et al., 2014). Previous studies have found that serum S100β, an astrocyte A1-specific marker, was a useful marker that correlated with several brain diseases, including SZ (Isobe-Harima et al., 2008; Langeh and Singh, 2021). Although serum BDNF as a biomarker for a variety of brain diseases is controversial, a meta-analysis found significant associations between lower serum BDNF and smaller hippocampal volumes in SZ (Ahmed et al., 2021), which suggests that serum BDNF may reflect the survival environment of neurons in the SZ brain to a certain extent. Moreover, since free PUFAs in the blood can pass the blood–brain barrier, fatty acid concentrations in the periphery can reflect the PUFAs level in the brain. Therefore, the present study measured the levels of plasma cytokines, glial markers, neurotransmitters, and erythrocyte membrane PUFAs to evaluate the changes of these factors and the relationships between the abovementioned several parameters in SZ.

### 2.3. Blood collection, processing, and separation of peripheral blood mononuclear cells

Venous blood was drawn into blood collection tubes containing EDTA from a forearm vein of participants between 7 and 9 a.m. following an overnight fast and then transferred to the laboratory and centrifuged immediately after arrival (4°C, 10 min, 3,000 rpm) within 3 h after the time of blood draw. The blood samples were stored at −80°C. Blood collection took place from March 2016 to March 2017.

Peripheral blood mononuclear cells (PBMC) were isolated by human peripheral blood lymphocyte separation solution (Shanghai Solarbio Biotechnology Co., Ltd, China), and then 90 μl of cell suspension (1.1 × 10^7^ cells/ml) was seeded to each well of 96-well plates. Following treatment with concanavalin A (ConA, Sigma, USA, final concentration 45 μg/ml) for 48 h, cell viability was measured with 3-(4, 5-Dimethylthiazol-2-yl)-2, 5- diphenyltetrazolium bromide (MTT, ATCC, USA) according to the manufacturer's instructions. The optical density was measured at 490 nm using a microplate reader (BioTek, USA). The absorbance of the control group was considered as 100% of cell proliferation.

### 2.4. The measurement of cytokine concentrations and glial markers by ELISA kits

Plasma levels of TNF-α, IL-1β, IL-2, IFN-γ, IL-4, IL-6, IL-10, IL-17, S100β, and BDNF were measured by commercially available enzyme-linked immunosorbent assay (ELISA) kits (Nanjing Jiancheng Bioengineering Institute, China), following the instructions provided by the manufacturer. The results were expressed as pg/ml of plasma.

### 2.5. Gas chromatography analysis of fatty acid profiles in erythrocytes

Fatty acid profiles in erythrocytes were valued by measuring n-3 PUFAs (including ALA, EPA, DPA, and DHA) and n-6 PUFAs (including LA, GLA, DGLA, and AA) using gas chromatography. Human erythrocytes were isolated from fresh anticoagulated blood samples after centrifuging at 2,500 r/min for 5 min. All erythrocyte samples were suspended in phosphate buffer (pH 7.4) for PUFA analysis. A 23:0 ethyl ester internal standard (100 μg/ml, NuCheck Prep, Elysian, MN) was added prior to extraction. PUFAs were extracted according to the method of Folch et al. (1957), using chloroform: methanol (2:1 v/v) with butylated hydroxytoluene as an antioxidant (Sigma-Aldrich, Bellefonte, PA) and subsequent addition of sodium phosphate. PUFA methyl esters were prepared by transesterification using 14% boron trifluoride in methanol (Thermo Scientific, Bellefonte, PA) with hexane on a 90°C heated block for 1 h (Morrison and Smith, 1964). They were then collected in hexane and analyzed on a Thermo Trace GC Ultra gas chromatograph (Thermo Scientific, USA) with an autosampler. A reference mixture of fatty acid standards (GLC-462, Nu-Chek Prep) was used for the assignment of retention times. Individual PUFA was expressed as the mole percentage of the total fatty acid pool, including both identified and unidentified peaks in the total fatty acid sum. PUFA concentrations were expressed as milligrams of fatty acids per gram of cellular proteins (mg/g) determined in the remaining cells, which were not utilized for fatty acid analysis.

### 2.6. Determination of membrane fluidity of erythrocytes by fluorescence anisotropy techniques

The fluidity of the erythrocyte membrane was evaluated by fluorescence polarization (P) and microviscosity (η). Anticoagulant blood samples were centrifuged at 2,500 r/min for 5 min to obtain erythrocytes in the lower layer. The cells were washed three times with PBS (5,000 rpm × 15 min), and then 10 mM Tris-HCI buffer solution (1:40, v/v) was added. After storing at 4°C for 60 min, the lysis solution was centrifuged at 150,000 rpm for 20 min to obtain erythrocyte membranes. Fluorescence probes 1,6-diphenyl-1,3,5-hexatriene (DPH, Sigma) dissolved in tetrahydrofuran were added to the suspension of the erythrocyte membrane with gentle mixing and incubated at 37°C for 30 min. The fluorescence anisotropy of the erythrocyte membrane was measured using the RF-540 fluorescence spectrophotometer (equipped with a polarization accessory provided by Beijing Science Instrument Factory). The excitation wavelength and the emission wavelength were 362 and 432 nm, respectively. The optical grating for both wavelengths was set at 5 nm. Four fluorescence intensities were measured for each sample. IVV: the fluorescence intensity measured when the optical axis of the polarizer and the polarizer are in the same vertical direction; IVH: the fluorescence intensity measured when the optical axis of the polarizer and the polarizer are in the vertical and horizontal directions, respectively; IHV: the fluorescence intensity measured when the optical axis of the polarizer and the polarizer are in the horizontal and vertical directions, respectively; and IHH: the fluorescence intensity measured when the optical axis of the polarizer and the polarizer are in the same horizontal direction for fluorescence intensity. Fluorescence intensity (I), correction factor (G), fluorescence anisotropy (P), and membrane fluidity (FLU) were calculated according to the following formulas: I = Ivv + 2IvH, G = IHv/IHH, P = (Ivv-G × IvH)/(Ivv + G × IvH), and FLU = 1/P.

### 2.7. Measurement of monoamine neurotransmitter and metabolite concentrations using high-performance liquid chromatography

The blood samples were centrifuged at 3,000 r/min for 10 min at 4°C, and plasma samples were obtained. The neurotransmitters and metabolites in the plasma were extracted with 0.5 M perchloric acid solution containing 0.5 mM EDTA-Na2. Following centrifugation at 14,000 *g* for 15 min at 4°C, the clear supernatant above the mixture was mixed with an equal volume of 1.5 M potassium phosphate dibasic solution containing 0.5 mM EDTA-Na2 and then centrifuged at 14,000 *g* for 15 min at 4°C. The clear supernatants were filtered through a 0.25-μm filter and stored at −70°C until further assays. The levels of dopamine (DA), serotonin (5-HT), norepinephrine (NE), and their metabolites (DOPAC; HVA, 5-HIAA, MHPG) were determined using liquid chromatography (HPLC). The HPLC system consisted of an analytical C18 reversed-phase column (ODS3 C18, 4.6 × 250 mm i.d., 5-micrometer particle size) and a fluorescence detector. The mobile phase consists of 0.02 M sodium acetate, 0.042 M methane sulfonic acid, and 0.1 mM EDTA, buffered to a pH of 4 with 0.0125 M citric acid. The flow rate was set at 1.0 ml/min, and the injection sample volume was 10 μL. The fluorescence detection was carried out with excitation wavelengths of 330 nm and emission wavelengths of 280 nm. The working standard solutions were freshly prepared on ice and stored at −20°C before use. Neurotransmitters and metabolites were identified by comparing the retention time of each peak in the sample solution, where each individual peak was further compared to the standard solution of DA, 5-HT, NE, and their metabolites (Sigma-Aldrich, USA), which served as an internal standard.

### 2.8. Statistical analysis

Statistics were performed using IBM SPSS Statistics 22 for Windows. Results are expressed as mean ± standard deviation (SD) or median and range. Quantile–quantile (Q–Q) plots and the Shapiro–Wilk test were used to assess the normality of variable distributions. Normally distributed data were analyzed using the parametric procedures Student's *t*-test or ANOVA and Pearson's correlation analysis. Non-normally distributed data were analyzed using the Mann–Whitney test or chi-squared (χ^2^) test and Spearman's correlation analysis. Two-tailed tests were used for all statistical analyses, and a *p*-value of < 0.05 was considered statistically significant.

## 3. Results

### 3.1. Increased lymphocyte proliferation and proinflammatory cytokines in schizophrenia

**Figure 2:**
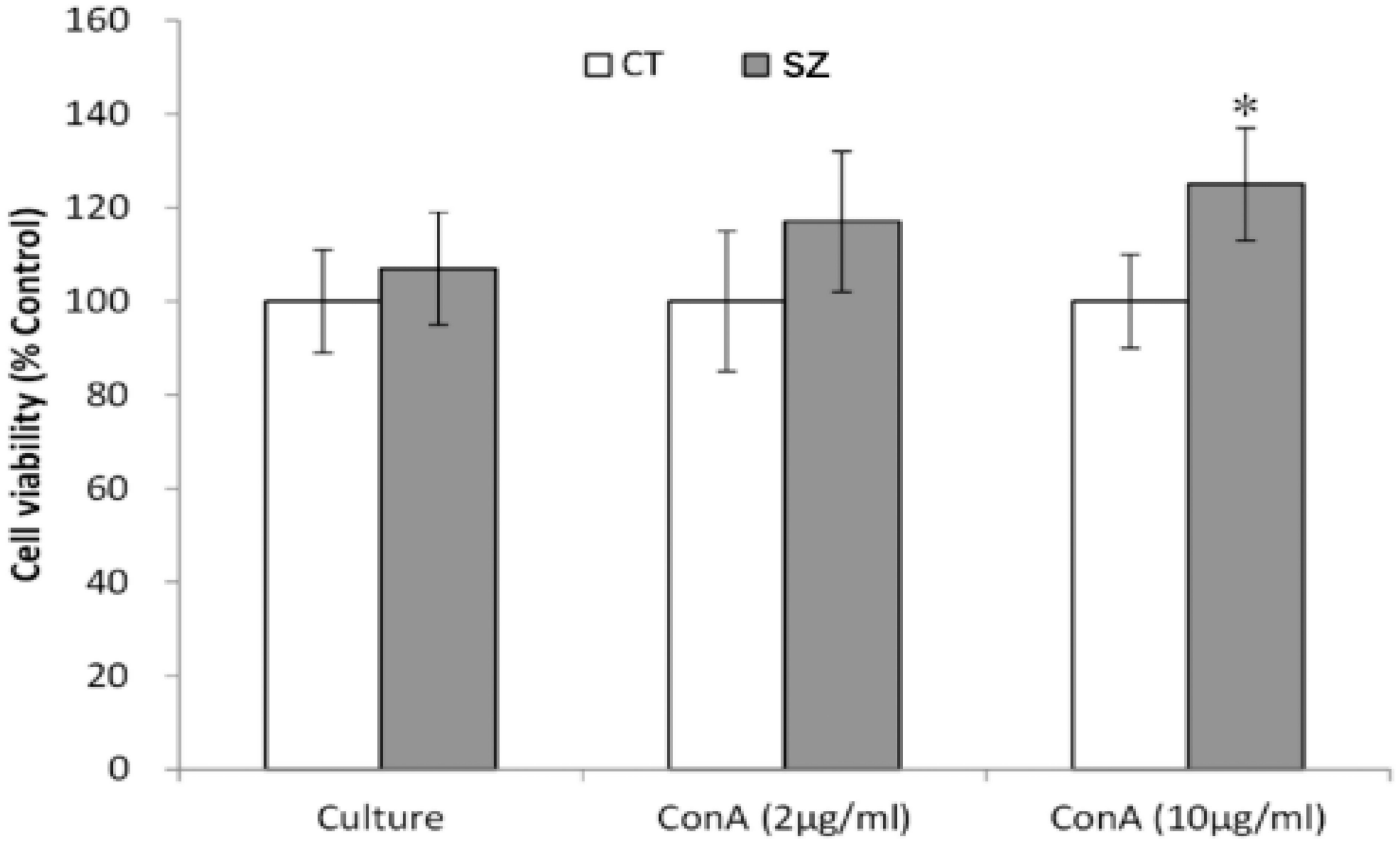
Proliferation ability of PBMC. CT, controls; SZ, schizophrenia patients, *p < 0.05 vs. control group.

As shown by the MTT assay, lymphocyte proliferation was significantly increased in SZ after stimulation with Con A at 10 μg/ml (*p* < 0.05) when compared to the control group (Figure 2).

Furthermore, a significantly higher level of Th1-released cytokine TNF-α (*p* < 0.05) and Th2 cell-released cytokine IL-6 (*p* < 0.01) was found in the plasma of SZ than controls, while a lower level of Th17 cells-released cytokine IL-17 (*p* < 0.05) was found only in the plasma of female SZ. However, no significant difference was found between SZ and healthy controls for other Th1 and Th2 cytokines (Table 2).

**Table 2:** Concentration of Th subtype cytokines and glia phenotype markers in the plasma (pg/ml).

**Groups**		**CT**	**SZ**
Th1	IL-2	153.93 ± 21.36	127.28 ± 16.23
	IFN-γ	13.52 ± 1.64	16.53 ± 3.85
	TNF-α	4.10 ± 0.23	5.28 ± 0.35^*^
Th2	IL-4	47.78 ± 9.69	39.75 ± 3.14
	IL-6	1.31 ± 0.16	3.54 ± 1.01^**^
	IL-10	119.24 ± 17.25	137.12 ± 19.34
Th17	IL-17 (F)	3.95 ± 0.34	3.11 ± 0.20^*^
M1	TNF-α	4.10 ± 0.23	5.28 ± 0.35^*^
	IL-1β	5.41 ± 0.40	7.03 ± 0.46^*^
	IL-6	1.31 ± 0.16	3.54 ± 1.01^**^
M2	IL-4	47.78 ± 9.69	39.75 ± 3.14
A1	S100B	229.89 ± 30.91	348.44 ± 42.08^*^
A2	BDNF	17195.71 ± 1772.59	10289.92 ± 1182.83^**^

CT, controls; SZ, schizophrenia patients; F, females. ^*^p < 0.05, ^**^p < 0.01, vs. the control group.

### 3.2. Imbalanced glia phenotypes and the correlations with cytokines

**Figure 3:**
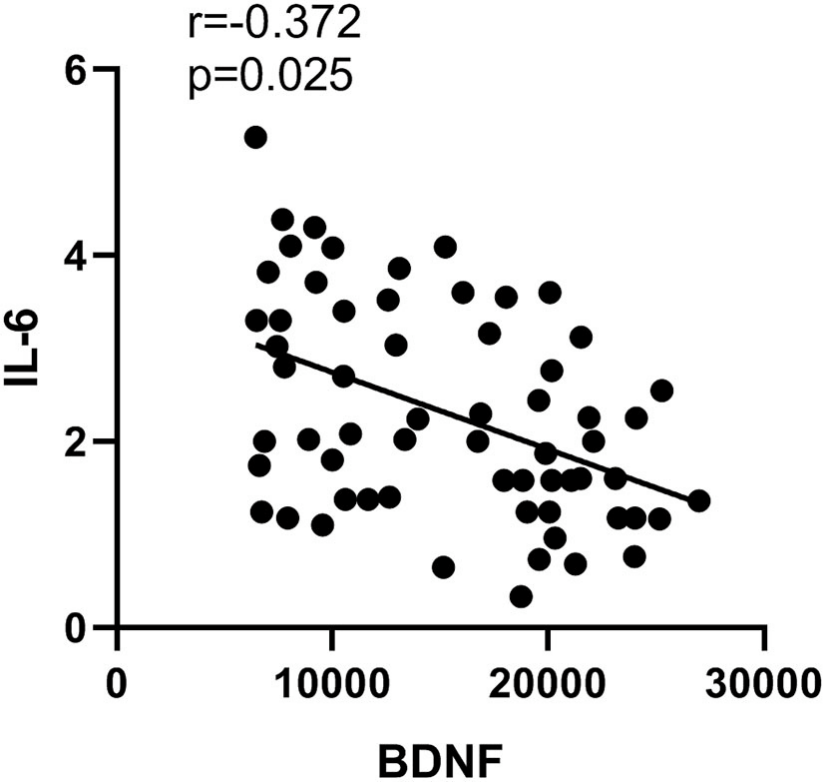
Correlation between the content of IL-6 and BDNF.

Compared to healthy controls, microglia M1 markers TNF-α, IL-1β, and IL-6 were all significantly higher in the plasma of SZ (*p* < 0.05 or *p* < 0.01). However, there were no significant changes in microglia M2 marker IL-4 in SZ when compared to healthy controls. Moreover, the level of astrocyte A1 maker S100B in SZ was significantly higher (*p* < 0.05), while the level of astrocyte A2 maker BDNF was significantly lower (*p* < 0.01) than those in the controls (Table 2). Moreover, IL-6 was negatively correlated with astrocyte phenotype A2 marker BDNF (*p* = 0.03, *r* = −0.37, Figure 3).

### 3.3. Decreased n-3 PUFAs in SZ and the correlation with cytokines

n-3 PUFA contents of EPA, DPA, and DHA were all significantly lower in SZ compared to healthy controls (*p* < 0.01 or < 0.05, Table 3). However, there was no difference in membrane lipids LA, GLA, ALA, AA, and DGLA concentrations between patients and controls. Overall, the patients with SZ showed a significantly lower level of total n-3 PUFAs (*p* < 0.01), which caused n-3/n-6 PUFA ratios in the SZ group to be significantly lower (*p* < 0.01) than that in the healthy controls (Table 3).

**Table 3:** Fatty acid concentrations in erythrocyte membranes (mg/g).

**Groups**	**CT**	**SZ**
LA	242.34 ± 19.37	239.74 ± 20.23
GLA	10.21 ± 1.01	9.51 ± 0.72
ALA	14.53 ± 1.14	15.37 ± 1.13
DGLA	21.62 ± 2.32	23.02 ± 2.36
AA	218.75 ± 12.99	234.03 ± 17.64
EPA	41.73 ± 3.67	20.17 ± 2.04^**^
DPA	30.21 ± 1.72	19.92 ± 1.12^*^
DHA	107.32 ± 7.95	76.82 ± 4.27^*^
Total n-6	491.64 ± 37.35	502.16 ± 46.32
Total n-3	152.72 ± 14.51	102.35 ± 11.42^**^
n-3/n-6	0.36 ± 0.03	0.23 ± 0.03^**^

CT, controls; SZ, schizophrenia. ^*^p < 0.05, ^**^p < 0.01, vs. controls.

**Figure 4:**
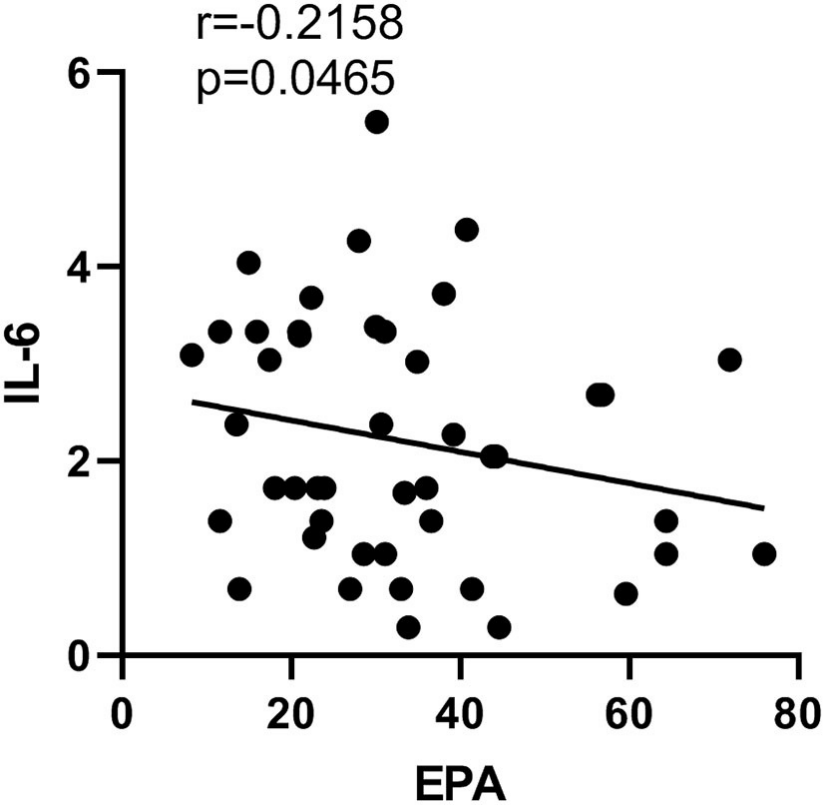
Correlation between EPA in erythrocyte and cytokine concentration IL-6 in SZ.

EPA concentration in erythrocytes was negatively correlated with the plasma level of IL-6 (*p* = 0.047, *r* = −0.22), while no correlation between any other PUFAs and cytokines was found in the present study (Figure 4).

### 3.4. Abnormal metabolism of neurotransmitters and the correlations with cytokines or PUFA contents

**Figure 5:**
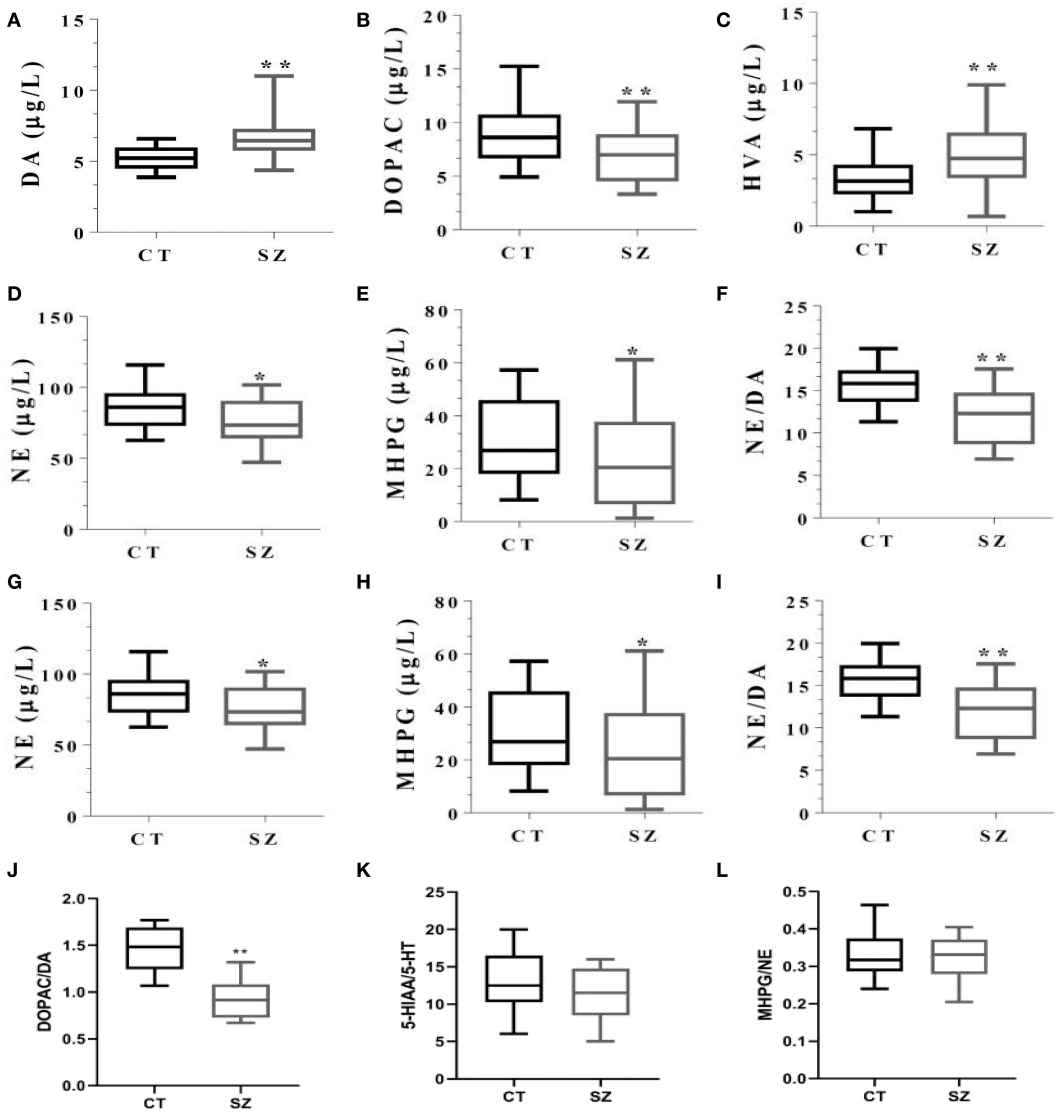
Levels of monoamine neurotransmitters and their metabolites in plasma of SZ and controls. CT, controls; SZ, schizophrenia, **(A)** DA, **(B)** DOPAC, **(C)** HVA, **(D)** 5-HT, **(E)** 5-HIAA, **(F)** 5-HT/DA, **(G)** NE, **(H)** MHPG, **(I)** NE/DA, **(J)** DOPAC/DA, **(K)** 5HIAA/5-HT, **(L)** MHPG/NE. *p < 0.05, **p < 0.01, vs. controls.

Compared to the control, plasma DA concentration and its final metabolite HVA were higher (both *p* < 0.05), while both its intermediary metabolite DOPAC and DOPAC: DA ratios were lower in SZ (*p* < 0.05 or *p* < 0.01, Figures 5A–C, J, respectively). Moreover, 5-HT concentration and its metabolite 5-HIAA, NE, and its metabolite MHPG, NE/DA ratio, and the 5-HT/DA ratio were all significantly smaller in SZ than those in the controls (*p* < 0.05 or *p* < 0.01, Figures 5D–I). However, there were no significant changes in ratios of 5HTAA/5HT or MHPG/NE between the two groups (Figures 5K, L).

**Figure 6:**
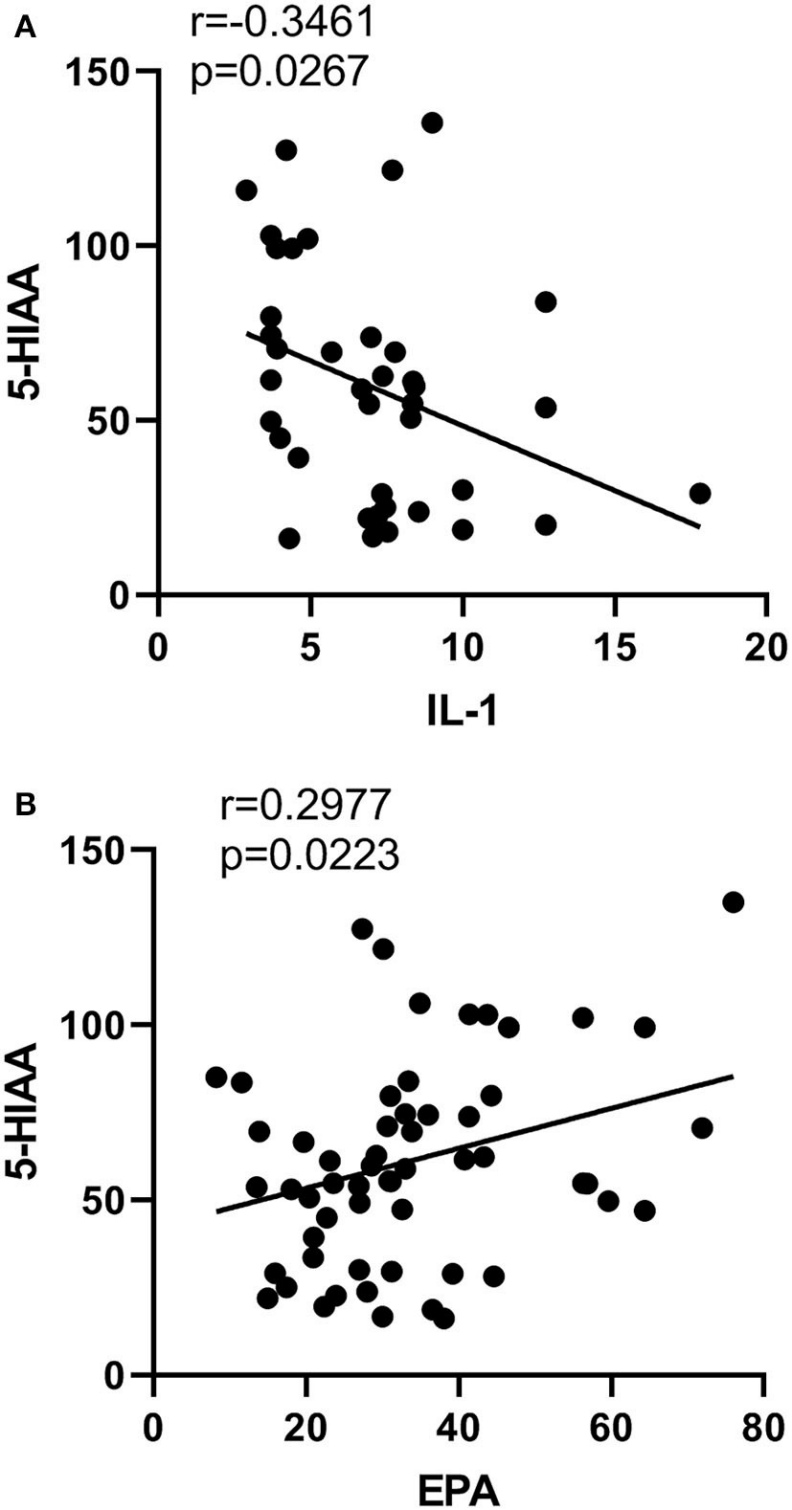
Correlations between neurotransmitters and cytokines or PUFA concentrations. Correlations **(A)** between the content of IL-1β and 5HIAA, **(B)** 5HIAA and EPA.

IL-1β was negatively correlated with 5HIAA (*p* = 0.03, *r* = −0.35, Figure 6A). Moreover, EPA concentration in erythrocytes was positively correlated with 5-HIAA (*p* = 0.02, *r* = 0.30, Figure 6B). However, there was no correlation between any other neurotransmitters and cytokines, or between any other PUFAs and the neurotransmitters in SZ.

### 3.5. Decreased membrane fluidity and correlations with PUFAs, cytokines, or neurotransmitters

**Figure 7:**
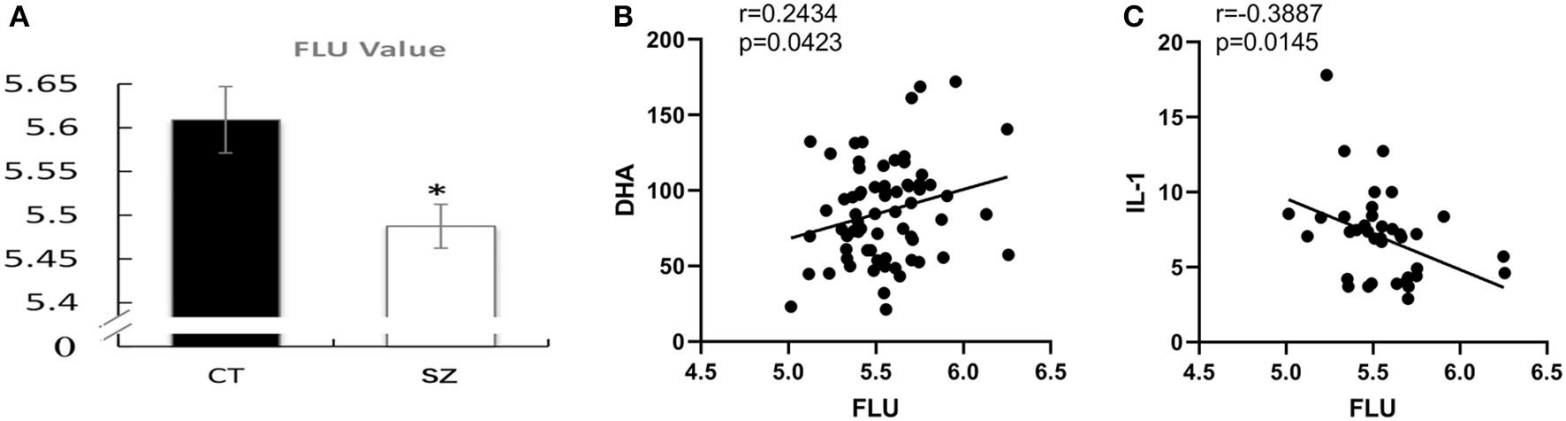
Decreased membrane fluidity and correlations with PUFAs or cytokines. **(A)** Erythrocyte membrane fluidity in SZ and controls, CT, control; SZ, schizophrenia, *p < 0.05 vs. controls. Correlations between **(B)** erythrocyte FLU and DHA concentration, **(C)** FLU and IL-1β.

Compared to the healthy controls, the FLU value was significantly decreased (*p* < 0.05) in the erythrocyte membrane of the subjects with SZ (Figure 7A).

Moreover, FLU was positively correlated with DHA content (*p* = 0.04, *r* = 0.24) while was negatively correlated with pro-inflammatory cytokine IL-1β (*p* = 0.01, *r* = 0.39, Figures 7B, C). However, there was no correlation between membrane fluidity and any other PUFAs, cytokines, or neurotransmitters.

## 4. Discussion

To explore the correlations between omega-3 fatty acids and inflammatory/glial abnormalities in schizophrenia, the present study demonstrated an inflammation-dominant immunity, but an unclear Th pattern in SZ. Furthermore, individuals with SZ showed a lower n-3 PUFA concentration and a higher ratio of n-6/n-3 PUFAs in lipid composition, lower membrane fluidity, and abnormal neurotransmitter and metabolite concentrations when compared to health controls. Furthermore, Pearson's analyses revealed that (1) elevated proinflammatory cytokine IL-6 was correlated with decreased A2 marker BDNF and decreased EPA, while increased IL-1β was correlated with decreased 5HIAA; (2) decreased n-3 PUFA EPA was correlated with decreased 5HIAA; and (3) decreased FLU was correlated with increased IL-1β and decreased DHA. Several findings gained in the present study are discussed below.

### 4.1. No clear Th1 and Th2 pattern was found in SZ

Because mitogen Con A mainly stimulates T lymphocyte proliferation, the increased lymphocyte proliferation may indicate some T lymphocyte activation in SZ. Thus, the study measured cytokines released by different subtypes of T lymphocytes. Th-1 cytokines produced TNF-α was significantly increased in SZ, which was consistent with a previous study (Kim et al., 2009). However, no significant difference for the other Th1 cytokines (IFN-γ and IL-2) was found between SZ and healthy controls in the present study. Since macrophage/microglia M1 phenotype and Th17 can also release TNF-α, increased TNF-α cannot reflect a Th1 pattern. Therefore, no clear Th1 pattern appeared in SZ patients. Furthermore, the present study found that one of the Th2 cytokines IL-6 was significantly increased in the plasma of SZ. High expression of IL-6 in childhood was found to increase the risk of SZ in adulthood (Khandaker et al., 2014). As a pro-inflammatory cytokine, IL-6 can induce neuronal apoptosis through the caspase-1/IL-18 pathway (Jha et al., 2010). Even though IL-6 is referred to as a controversial marker of the Th-2 immune response, it can be produced by other kinds of activated immune cells, such as macrophages (Andres-Rodriguez et al., 2020). Since no significant difference for two other Th2 cell-releasing cytokines (IL-4 and IL-10) occurred between SZ and healthy controls, the present study could not confirm the Th2 shift in SZ. Therefore, there is possibly no specific Th pattern shift in SZ. Interestingly, the present study found Th17 released cytokine IL-17 was decreased in the plasma of female patients but not in male patients with SZ. A recent study has shown that IL-2 serum levels were lower in the female group than in the male group after chronic clozapine treatment, which indicates a gender difference in the abnormal expression of cytokines between male and female patients with SZ (Yuan et al., 2022). Since serum IL-17 was found to promote Th-1 cell response through modulating dendritic cell function (Gottenberg and Chiocchia, 2007), decreased IL-17 seems consistent with the inactivation of Th-1 in SZ observed by this study. Furthermore, plasma levels of Th17-related cytokines were found to correlate with aggressive behavior in patients with SZ (Li et al., 2016), which is consistent with less aggressive behavior in female SZ patients reported by Chen and Zhou (2012).

### 4.2. Imbalanced glia phenotypes and their relationship with cytokines

The present study found that pro-inflammatory cytokines from macrophage/microglia M1, including TNF-α, IL-1β, and IL-6, were significantly increased in the plasma of SZ, which was consistent with previous studies (Kim et al., 2009). Since no significant change in macrophage/microglia M2 marker IL-4 was found in SZ, an imbalance between microglia M1 and M2 is toward enhanced inflammatory response in SZ.

Since S100β is considered an astrocyte A1 marker, increased S100β in SZ plasma observed by the current study may suggest activation of the A1 phenotype in SZ. Changes in plasma S100β concentration have been reported as an index of several brain diseases, including SZ (Isobe-Harima et al., 2008; Langeh and Singh, 2021). Increased S100β has been found to accelerate neuroinflammation, which leads to neurotransmitter dysfunction and neuronal damage (Sorci et al., 2013). By contrast, an astrocyte A2 phenotype marker BDNF was decreased in the plasma of patients. The main functions of BDNF are survival-promotive on a variety of neurons, including hippocampal and cortical cholinergic, nigral dopaminergic, and serotonergic neurons (Angelucci et al., 2005). The imbalance between astrocyte A1 and A2 may result from the imbalance between microglia M1 and M2. Moreover, for the first time, it has been shown that IL-6 is negatively correlated with A2 marker BDNF in the present study. This may indicate that increased M1 and related inflammatory response may contribute to neuronal apoptosis and neurogeneration in SZ.

### 4.3. Decreased n-3 PUFAs in SZ and their correlation with cytokines

Since blood flows through various organs and tissues in the body, including brain tissues, changes in blood PUFA concentration can affect the function of various organs and cells. Previous studies have reported deficient n-3 PUFA composition in the blood or erythrocyte membranes of SZ patients (Horrobin et al., 1994; Nuss et al., 2009). Moreover, the n-3 PUFA content was used to predict the outcome of SZ treatments (Sumiyoshi et al., 2011). Since free fatty acids in the blood can pass the blood–brain barrier, the composition of fatty acids in erythrocyte membranes and the membrane fluidity can reflect the membrane lipid composition and fluidity in the brain to a certain extent. Therefore, this study selected erythrocytes that are relatively easy to obtain as the target cells to study the lipid composition and membrane fluidity. The present study further demonstrated that n-3 PUFAs (including EPA, DPA, and DHA) were lower in SZ when compared to healthy controls. Thus, the ratio of n-3/n-6 in the SZ was inevitably decreased. A decreased n-3/n-6 PUFA ratio may lead to a high risk of inflammatory response (James et al., 2000). Indeed, plasma levels of proinflammatory cytokines TNF-α, IL-1β, and IL-6 were all significantly increased when compared to the healthy controls. Furthermore, the present study confirmed a negative correlation between n-3 PUFA (EPA) and pro-inflammatory IL-6 in SZ. This result is in line with previous studies that found a negative correlation between EPA and IL-6 or TNF-α levels in patients with bipolar disorder (Koga et al., 2019). Recently, a clinical study also revealed that the baseline plasma concentrations of inflammatory markers were negatively associated with baseline omega-3 index in SZ (Susai et al., 2022). However, curiously, there is no significant link between n-3 PUFA DHA and any cytokines. Consistently, our previous results showed a stronger anti-inflammatory effect of pure EPA or higher EPA combined with DHA than DHA or lower EPA combined with DHA in *in vitro* experiments (Zhang et al., 2018). Taken together, our findings provide evidence that EPA was clinically effective in attenuating SZ symptoms but not DHA (Smedslund and Berg, 2011). However, considering the heterogeneity of SZ, the beneficial effect of EPA may only be targeted at a certain subset of SZ patients. For example, in a randomized controlled trial EPA 2 g/day alone was found to be detrimental for a subgroup of SZ (“low PUFA,” vs. “high PUFA”) (Bentsen et al., 2011, 2013). Therefore, the effect of EPA on SZ patients may require further classification.

### 4.4. Abnormal metabolism of neurotransmitters and the relationship with cytokines

In the present study, plasma levels of DA and its final metabolite HVA in SZ were higher, while its intermediary metabolite DOPAC was lower than those in the controls, confirming the abnormal dopaminergic metabolism in SZ. A previous study reported that immunostimulation during pregnancy increased the number of dopaminergic neurons in the fetal brain (Winter et al., 2009). Moreover, proinflammatory cytokines can also increase the amount of rat mesencephalic progenitor cells and promote their transformation into dopaminergic neurons (Potter et al., 1999). Thus, changes in the DA system may result from increased macrophage/microglia M1 phenotype and related proinflammatory cytokines, including TNF-α, IL-1β, and IL-6. With regard to other monoamine neurotransmitters, the current study found that both 5-HT and its metabolite 5-HIAA and NE and its metabolite MHPG were decreased, and both the NE/DA ratio and the 5-HT/DA ratio were significantly lower in SZ than in healthy controls, which indicate that both metabolisms of 5-HT and NE are low and insufficient, while DA function is hyperactive in SZ. The imbalances between DA and other neurotransmitters were supposed to be related to the neuropathological changes in SZ, such as “negative” symptoms and cognitive decline (Meltzer, 1989). From a psychoneuroimmunologic point, a negative correlation between IL-1β and 5HIAA was found in the present study. It is well known that on the one hand, proinflammatory cytokines degrade tryptophan via activating indoleamine 2,3-dioxygenase (IDO), which reduces serotonin synthesis (Schiepers et al., 2005). On the other hand, proinflammatory cytokine activated catechol-O-methyltransferase (COMT) and monoamine oxidase (MAO) to increase 5-HT metabolism (Wang et al., 2001). This may result in a positive correlation between IL-1β and 5-HT metabolite 5-HIAA (Linthorst et al., 1995). Deficiency in serotonergic and noradrenergic neurotransmission has been associated with low-grade neuroinflammation, which was attenuated by anti-inflammatory compounds in SZ, as shown in meta-analyses (Cakici et al., 2019). In fact, the modulation of neurotransmitter systems operated by anti-psychotic treatments was able to normalize cytokine productions and, consequently, Th1/Th2 balance (Song et al., 2000).

### 4.5. The relationship between PUFAs and neurotransmitter concentrations

The present study for the first time showed that the concentration of EPA in erythrocytes was positively correlated with 5-HIAA, while there was no significant correlation between DHA and any neurotransmitter. The important finding that the EPA level is more related to the abnormal neurotransmitter system than DHA in SZ further revealed the reason why EPA more effectively improved symptoms of mental illness, such as depression and SZ in clinical trials, but not DHA or any other PUFA (Hashimoto et al., 2014).

### 4.6. Decreased membrane fluidity and correlations with PUFAs, cytokines, or neurotransmitters

Decreased membrane fluidity implicates the etiology of psychiatric and neurological diseases. As mentioned above, PUFAs can modulate membrane-bound protein functions, including ion channels, receptors, and enzymes (Wang et al., 2002). The incorporation of DHA and EPA into membrane phospholipids, such as phosphatidylcholine, phosphatidylethanolamine, and phosphatidylserine, can markedly reduce membrane bilayer thickness and increase membrane fluidity (Song et al., 2016). In addition, n-3 PUFAs can reduce cholesterol content or cholesterol/phospholipid ratio, which also increase membrane fluidity (Hashimoto et al., 1999). Increased membrane fluidity may enhance cell migration, proliferation, differentiation, and protein and gene interaction (Noutsi et al., 2016). Moreover, the formation of membrane microdomains, such as lipid rafts and caveolae that play the role of signaling platforms, is also highly related to membrane fluidity (Mollinedo and Gajate, 2015). Interestingly, the present study for the first time demonstrated that decreased membrane fluidity in SZ was positively correlated with decreased DHA contents. DHA is the end product of EPA and DPA. As the highest content n-3 PUFA in the cell membrane, the decreased DHA may reduce the content of total n-3 PUFA in cell membranes and the membrane fluidity. Since most cellular functions depend on the membrane structure and fluidity, the decreased n-3 PUFAs as well as membrane fluidity may be one cause of neuronal dysfunction in SZ.

More interestingly, the present study for the first time found membrane fluidity was negatively correlated with proinflammatory cytokine IL-1β. It should be noted that IL-1β negatively correlated with FLU, while IL-6 is correlated with BDNF and EPA in the present study. Studies have reported that an inflammatory response induced by IL-1β could reduce n-3 fatty acid contents (Taepavarapruk and Song, 2010), which results from the competition of n-6 fatty acids, precursors of proinflammatory factors. The inflammatory response and changed PUFA composition of the cell membrane may reduce membrane fluidity (negative correlation between IL-1 and FLU). It is well known that BDNF plays important roles in neurogenesis and depression. It was reported that IL-6 cytokine increased neural stem cell proliferation and astrogliogenesis but decreased neurogenesis (Wang et al., 2011). Moreover, the plasma levels of BDNF were higher, whereas IL-6 was lower in epilepsia patients than controls without changing IL-1 (Alvim et al., 2021). The evidence suggests a possible relationship between IL-6 and BDNF. However, IL-1 has not been reported to have such a relationship with BDNF. Our previous studies have demonstrated that EPA and its metabolites can reduce both IL-1 and IL-6 concentrations as well as inflammatory responses. However, it is unclear whether EPA directly or through its metabolites inhibits inflammation to exert its anti-inflammatory effects on different cytokines. Even though there is a certain relationship between EPA and inflammatory factors, the correlation between EPA and IL-6 is unclear, which needs to be further studied.

Other limitations in the present study are that first, some patients were smoking before hospitalization, which may affect their immune function and inflammation levels. This requires us to collect more no-smoke cases or find a better method to eliminate this interference; second, a lower level of education in SZ patients than health controls may cause certain differences in the experiment indicators in the present study. Thus, more matched controls should be recruited in future studies; third, compared to health controls, n-3 PUFA (including EPA, DPA, and DHA) levels were decreased in SZ in this study, which may be related to dietary habits or to hospital diets in the patients, the effects of which should be considered. Fourth, as we lacked data on PANSS, the correlation between fat acids and the severities of positive, negative, and general psychopathology symptoms in PANSS are not available. This may affect the possibility of using fatty acid content in the blood as a clinical symptom indicator. Fifth, since TNF-α and IL-6 are also produced by macrophages in the adipose tissue and muscle, information, such as obesity and physical activity in SZ patients should be included when the patient enters the hospital. Sixth, many other factors, including the disease stage (acute and chronic phase), the reaction to antipsychotic drugs, and the level of nicotine use, should also be considered as having a certain impact on the detected indicators. Finally, due to the heterogeneity of SZ and the small sample size in the present study, the findings may only be applicable to certain subgroups of SZ patients in China. Taken together, patients should be further divided into different subgroups based on different conditions in future studies.

Despite these limitations, the present study demonstrated that (1) no specific Th pattern shift occurred in SZ; (2) cytokine changes toward the pro-inflammatory pattern may be derived from an imbalance between macrophage/microglia M1 and M2, which lead to the activated astrocyte A1 phenotype; (3) increased pro-inflammatory cytokines were correlated with decreased n-3 PUFA and dysfunctional neurotransmitter systems; (4) EPA is more correlated with the abnormal neurotransmitter system than DHA; and (5) decreased membrane fluidity is correlated with increased pro-inflammatory cytokine IL-1β or decreased DHA in SZ. By highlighting the relationship among cytokines, fatty acids, and neurotransmitters, these findings may potentially shed light on the neuropathological mechanism of schizophrenia.

## Data availability statement

The raw data supporting the conclusions of this article will be made available by the authors, without undue reservation.

## Ethics statement

The studies involving humans were approved by the Research Ethics Committee for Biomedical Subjects, according to Zhujiang Hospital of South Medical University guidelines. The studies were conducted in accordance with the local legislation and institutional requirements. The participants provided their written informed consent to participate in this study.

## Author contributions

CS applied for the grant, directed and designed the experiments, and wrote the manuscript. YZ applied for the grant, performed the experiments, analyzed the data, and wrote the manuscript. JY collected blood samples of patients and health controls, analyzed the data, and wrote the manuscript. HY, JL, and LZ collected blood samples of patients and health controls. LY, YuL, CZ, YaL, BL, and XH performed the experiments. All authors contributed to the article and approved the submitted version.
